# A Robust Capon Beamforming Algorithm with Desired Signal Steering Vector Correction

**DOI:** 10.3390/s25154570

**Published:** 2025-07-24

**Authors:** Zhiqi Gao, Bowen Wu, Pingping Huang, Wei Xu, Weixian Tan, Zhixia Wu

**Affiliations:** 1 College of Information Engineering, Inner Mongolia University of Technology, Hohhot 010080, China; gzqngd@imut.edu.cn (Z.G.); hpp@imut.edu.cn (P.H.); xuwei1983@imut.edu.cn (W.X.); wxtan@imut.edu.cn (W.T.); zxwu@imut.edu.cn (Z.W.); 2 Inner Mongolia Key Laboratory of Radar Technology and Application, Hohhot 010051, China

**Keywords:** Capon beamforming, steering vector optimization, constraint parameter, covariance matrix reconstruction

## Abstract

The conventional Capon beamforming algorithm can achieve a high gain in the direction of desired signals and zero-trapping in the direction of interfering signals, providing a high output signal-to-interference-plus-noise ratio (SINR). However, when the steering vector of the desired signal is mismatched, the performance of the Capon beamforming algorithm degrades. In addressing this challenge, the present research introduces a refined algorithm. The core of the proposed robust Capon beamforming technique lies in leveraging the orthogonality between the steering vector and the noise space, the estimated expected signal steering vector is corrected. Based on this feature, the proposed algorithm meticulously optimizes the predicted steering vector of the desired signal, which can mitigate the problem of performance degradation caused by the mismatch in the steering vector. Moreover, the covariance matrix is corrected using the desired signal elimination method, which can overcome the problem of signal self-cancelation. Furthermore, through the optimization process, the proposed algorithm can maintain high robustness in complex environments and under the condition of different input signals, its beam pattern performance is more excellent. The results of simulation experiments show that the proposed algorithm demonstrates greater robustness compared to the currently available algorithms, can achieve a higher output SINR, and is insensitive to steering vector mismatch.

## 1. Introduction

Currently, adaptive beamforming is one of the key techniques in the field of array signal processing. It also plays an important role in various tasks, including wireless communication, radar, and sonar applications [1]. However, when multiple sensors in an array receive or send signals directionally, they need to optimize the pattern generation process based on the differences in the spatial paths during signal propagation and accordingly assign different antenna gains to signals in different arrival directions; this process is called beamforming. The adaptive beamforming method is representative of beamforming methods. This method can accurately focus narrow beams on useful signals in real time while suppressing side lobes in other directions, thus achieving directional reception, which helps improve the overall capacity of a system [2,3,4].

On the other hand, adaptive beamforming algorithms allow the patterns synthesized by sensors to be steered in some directions, and automatically formed the nulls in the direction of interference. The purpose of this is to suppress interference, thereby improving the signal-to-interference-plus-noise ratio (SINR). Capon [5] proposed the minimum variance distortionless response (MVDR) beamforming technique, which can effectively suppress interference by minimizing the total power of the array output signal while keeping the desired signal direction undistorted. The MVDR beamforming technique can provide good directional resolution and interference suppression. However, it is highly sensitive to errors, and its performance can degrade when the steering vector shows a certain error. In practice, the existence of various parameter mismatches and systematic errors, such as the deviation in an array’s response signal from the expected one, can affect the interference suppression performance and cause self-cancelation of the helpful signal [6]. Therefore, developing a robust adaptive beamforming technique that can cope with errors has been an important research direction in the field of array signal processing.

To improve the robustness of the Capon beamforming algorithm, researchers have proposed a diagonal loading strategy. In addition, the stability of the sample covariance inversion (SMI)-based beamforming technique can be significantly improved by adding specific values to the main diagonal of the sample covariance matrix [7,8,9,10,11]. A scaled feature matrix is commonly added to the sample covariance matrix to improve an algorithm’s robustness; however, some problems can arise in selecting acceptable loading values in real applications [7]. Some algorithms can modify the loading value automatically and maintain a good signal-to-noise ratio (SNR) at a small number of snapshots [8,9]. Nevertheless, when the input SNR value is low, the performance of these methods can dramatically decrease.

In recent years, many steering vector optimization techniques have been proposed to enhance the robustness of beamforming algorithms [12,13,14,15,16,17,18,19,20,21]. For example, in [12], the authors introduced an improved robust Capon beamforming algorithm, which can effectively avoid the covariance matrix inversion by directly performing singular value decomposition of the received data matrix. In addition, in [13], an innovative algorithm was proposed to constrain the steering vectors to predefined spherical or ellipsoidal surfaces, which could be determined by solving a quadratic programming problem. Aiming to reduce the computational burden brought by integration, in [14], the authors divided the integration region into grids, and the integration results of each grid were summarized, thus effectively reducing the computational burden. Because the expansion of the integration range can increase redundant signals, an additional constraint was introduced to limit the steering vectors to the uncertainty set, and the spherical integration was optimized to circular integration; however, this might reduce the interference suppression degrees of freedom [15]. The aforementioned studies have provided various innovative perspectives and ideas for improving beamforming algorithms’ robustness. In [16], the response vector is optimized by transforming a non-convex problem into a semidefinite programming problem, which can effectively improve the SINR, suppress side lobes, and reduce the false alarm probability. In [17], the rate allocation in wireless acoustic sensor networks is modeled as minimizing transmission energy consumption under constrained noise suppression, and the block diagonal structure of the noise correlation matrix is utilized to enable distributed computation. In [18], the performance degradation of traditional single uncertainty set methods is addressed by using multiple small uncertainty sets to cover large uncertainty regions and developing an iterative algorithm for solution. In [19], the issue of misaligned steering vectors is addressed by combining the structural risk minimization of support vector machines with diagonal loading techniques to construct an optimized model. In [20], an optimization model is constructed to maximize array output power, and two methods are proposed for reconstructing the interference plus noise covariance matrix. In [21], a convex optimization model is constructed to estimate the expected signal steering vector, and an improved projection method is designed to correct the mismatch in the nominal steering vector.

However, adaptive beamforming not only needs to solve the problem of inaccurate direction estimation caused by a mismatch in the steering vectors but also considers the effect of the covariance matrix. To this end, many methods were proposed for the covariance matrix reconstruction [22,23,24,25,26,27,28,29,30,31,32,33]. For example, a robust beamforming algorithm was proposed in [22], integrating weighted spatial smoothing and the steering vector estimation. This algorithm constructs the weighting matrix by specially dividing the subarrays; it also adopts the nesting method based on the weighting differences in autocorrelation and intercorrelation between the subarrays to obtain a more accurate covariance matrix. To address the problem of the coexistence of mobile interference and model error, in [23], the authors developed a robust adaptive beamforming algorithm, combining covariance matrix coning with steering vector estimation. This algorithm first weighs the covariance matrix, then uses the enhanced covariance matrix to estimate the actual steering vectors, and finally combines the enhanced covariance matrix and the estimated steering vectors to realize the beamforming. In [24], a circular uncertainty set constraint is used to reconstruct the Interference-plus-Noise Covariance Matrix (INCM) without the target. In [25], the orthogonality of sparse steering vectors is used to estimate interference power, and the INCM is reconstructed by projecting the sample covariance matrix. In [27], the integration interval is divided based on Capon power spectrum peaks, the number of interference sources is determined using feature decomposition, and then the INCM is reconstructed. In [28], the Gaussian–Chebyshev integral is introduced to efficiently reconstruct the INCM within the corrected azimuth sector. In [31,33], projection operations are used to eliminate target signal components while retaining interference features. Method 1 in [31] directly reconstructs Interference Covariance Matrix (ICM) using projection snapshots, while Method 2 constructs a convex optimization problem using the orthogonality between interference steering vectors and subspaces. In [33], the interference subspace is obtained by training data using feature decomposition, and the INCM is reconstructed to eliminate the influence of target signals.

In recent years, there have been many related studies on Angle-of-Arrival (AoA) estimation for adaptive beamforming at the physical layer [34,35,36,37,38]. In radar systems, AoA estimation helps determine the direction of a target, enabling target tracking and localization. For example, in [34], an adaptive beam control architecture based on real-time AoA estimation is proposed, introducing phase shifts in the local oscillator (LO) path via an improved Direct Digital Synthesis-Phase-Locked Loop (DDS-PLL) structure to achieve dynamic beamforming in millimeter-wave communications. This method utilizes real-time AoA estimation to dynamically adjust the antenna radiation pattern, precisely targeting the target location to counteract millimeter-wave path loss and enhance antenna system gain. Performance verification is conducted via hardware-in-the-loop (HIL) simulation, with results demonstrating the architecture’s effective implementation of adaptive beam steering. In [35], three power-based AoA estimation algorithms compliant with 3GPP protocols are proposed and optimized to address beamforming requirements in millimeter-wave 5G NR systems. The modifications to the hierarchical search algorithm were proposed to overcome discretization error issues. The algorithm is numerically simulated using an actual ray-tracing channel model, meeting millisecond-level real-time requirements and low computational overhead, providing a highly reliable solution for 5G NR analog beamforming. In [36], a full-hardware method for AoA estimation in phased array antennas is proposed. This method adopts a modular structure, consisting of an analog part for radio frequency (RF) signal up/down conversion and a digital part responsible for AoA estimation. It calculates the phase difference to determine the AoA, which can be used for adaptive beamforming to extend the operational range or reduce transmission power in internet of things (IoT) applications. In [37], a new technique for estimating the AoA of multiple sources via phase interferometry is presented. Based on the in-phase/quadrature low-pass mixing (IQ LPM) algorithm, this technique obtains phase differences after low-pass filtering and eliminates phase ambiguities using the most significant bit (MSB) of the Q branch. In [38], a deep active learning scheme for multi-source AoA tracking in millimeter-wave arrays is proposed, which significantly enhances the multi-source AoA tracking performance in both uniform linear array (ULA) and uniform rectangular array (URA) scenarios.

Currently, traditional beamforming algorithms face numerous challenges in practical applications, such as, it is susceptible to steering vector mismatch and has poor interference suppression capability. To address these challenges, this study proposes a robust Capon beamforming algorithm. The algorithm constructs a Lagrangian function based on known parameters and dynamically optimizes constraint parameters within the estimated desired signal angle domain. Specifically, each estimated angle corresponds to a constraint parameter, which in turn corrects the steering vector of the desired signal. Combined with the modified covariance matrix, the optimal weight vector is determined. The algorithm maintains a high output SINR under varying input SNR, snapshot counts, multiple interferers, and angle deviations. This significantly addresses the lack of robustness and flexibility in many existing algorithms.

The remainder of this paper is organized as follows. Section 2 constructs a uniform linear array model and obtains the received array signal. Subsequently, Section 3 analyzes the optimization problem based on the optimal weight vectors of the Capon beamforming algorithm. Based on this, the beamforming algorithm with desired signal steering vector correction is proposed. Section 4 presents simulation results and performance analysis of the proposed algorithm. Finally, Section 5 concludes the paper.

## 2. System Modeling and Problem Analysis

Suppose that there are (*M* + 1) uncorrelated narrowband signals containing a desired signal and *M* interferences, which incident into a uniform antenna array consisting of *N* elements, where *N* > (*M* + 1). Then, the received signal can be expressed as follows:

(1) $$\begin{array}{cc} X(t) & =AS(t)+n(t)\\ & =a(\theta_{0})s_{0}(t)+\sum_{k=1}^{M}a(\theta_{k})s_{k}(t)+n(t), \end{array}$$

where ***A*** = [***α***(*θ*_0_), ***α***(*θ*_1_), …, ***α***(*θ_M_*)] is the matrix of steering vectors with dimensions of *N* × (1 + *M*); ***α***(*θ*_0_) is the desired signal steering vector, ***α***(*θ_k_*), where *k* = 1, 2, …, *M*, is the steering vector of the *k*th interference; ***S***(*t*) is the complex amplitude of the desired signal and interference; and ***n***(*t*) is the noise vector with dimensions of *N* × 1.

The covariance matrix of a received signal ***X***(*t*) can be obtained by

(2) $$\begin{array}{cc} R & =E[X^{H}(t)X(t)]\\ & =R_{S}+R_{J}+R_{N}\\ & =\sigma_{0}^{2}\alpha (\theta_{0})\alpha^{H}(\theta_{0})+\sum_{k=1}^{M}\sigma_{k}^{2}\alpha (\theta_{k})\alpha^{H}(\theta_{k})+\sigma_{n}^{2}I, \end{array}$$

where ***R****_S_*, ***R****_J_*, and ***R****_N_* denote the covariance matrices of the desired signal, interference, and noise, respectively; *σ*_0_^2^ is the desired signal power; *σ_k_*^2^ is the power of the *k*th interference; *σ_n_*^2^ is the noise power; ***I*** is the unit matrix; and [·]^H^ indicates the conjugate transpose operation.

The covariance matrix of the received signal cannot be obtained directly but is estimated from snapshot data as follows:

(3) $$\hat{R}=\frac{1}{K}\sum_{k=1}^{K}X(k)X^{H}(k),$$

where *K* denotes the number of snapshots, and ***X***(*k*) is the *k*th snapshot received by the array.

The design principle of the Capon beamformer is to minimize the output noise, containing the generalized noise, such as interference, as well as variance, while providing a distortion-free output of the signal in the direction of interest. The constraint that needs to be satisfied for a distortion-free output defines that the inner product of the weight vector and the steering vector of the desired signal should equal 1. Therefore, Capon beamforming represents an optimization problem with the following constraint:

(4) $$\left\{\begin{array}{l} \min W^{H}\hat{R}W,\\ s.t.W^{H}\hat{\alpha }(\theta_{0})=1, \end{array}\right.$$

where $\hat{\alpha }(\theta_{0})$ denotes the assumed desired signal steering vector, and ***W*** is the weight vector obtained by the sampling matrix inverse method as follows:

(5) $$W=\frac{{\hat{R}}^{-1}\hat{\alpha }(\theta_{0})}{{\hat{\alpha }}^{H}(\theta_{0}){\hat{R}}^{-1}\hat{\alpha }(\theta_{0})}.$$

The SINR value of the array output is calculated by

(6) $$SINR=\frac{W_{\mathrm{opt}}^{H}R_{S}W_{\mathrm{opt}}}{W_{\mathrm{opt}}^{H}R_{J+N}W_{\mathrm{opt}}},$$

where ***W***_opt_ is the optimal weight vector of beamforming.

The implementation of the above-presented Capon beamforming algorithm requires accurate knowledge of the signal steering vector. However, due to the presence of various errors, the assumed desired signal steering vector may mismatch with the true signal steering vector, which can further result in severe degradation of the algorithm’s performance and output SNR. To cope with the above-mentioned problems, this study develops a robust Capon beamforming algorithm.

## 3. Robust Capon Beamforming Algorithm

The proposed robust Capon beamforming algorithm includes two main mechanisms: steering vector correction and covariance matrix reconstruction. The steering vector correction improves the robustness, anti-interference ability, and estimation accuracy of the algorithm, and the covariance matrix reconstruction enhances the algorithm’s adaptability to dynamic environments and non-stationary signals. These two mechanisms jointly ensure that the beamforming algorithm can maintain excellent performance in complex environments.

### 3.1. Steering Vector Correction

In practice, the steering vector ***α***(*θ*_0_) of the desired signal cannot be obtained accurately [39]. Namely, ***α***(*θ*_0_) is restricted to the elliptic uncertainty set using known conditions, and it is estimated by a robust cone quadratic optimization algorithm as follows:

(7) $$\left\{\begin{array}{l} \max\;\sigma^{2},\\ s.t.\;\;R-\sigma^{2}\alpha^{H}(\theta_{0})\alpha (\theta_{0})\geq 0,\\ {\left(\alpha (\theta_{0})-\hat{\alpha }(\theta_{0})\right)}^{H}C^{-1}(\alpha (\theta_{0})-\hat{\alpha }(\theta_{0}))\leq 1, \end{array}\right.$$

where *σ*^2^ is the signal power, ***C*** is a given parameter matrix, $\alpha (\theta_{0})-\hat{\alpha }(\theta_{0})$ is the difference between the orientation vector and its estimated value.

By substituting Equation (5) into Equation (4), an estimation of *σ*^2^ can be obtained as follows:

(8) $$\begin{array}{l} \left\{\begin{array}{cc} W^{H}\hat{R}W & =(\frac{{\hat{\alpha }}^{H}(\theta_{0}){\hat{R}}^{-H}}{{\hat{\alpha }}^{H}(\theta_{0}){\hat{R}}^{-1}\hat{\alpha }(\theta_{0})})\hat{R}(\frac{{\hat{R}}^{-1}\hat{\alpha }(\theta_{0})}{{\hat{\alpha }}^{H}(\theta_{0}){\hat{R}}^{-1}\hat{\alpha }(\theta_{0})})\\ & =\frac{{\hat{\alpha }}^{H}(\theta_{0}){\hat{R}}^{-1}\hat{\alpha }(\theta_{0})}{{\left({\hat{\alpha }}^{H}(\theta_{0}){\hat{R}}^{-1}\hat{\alpha }(\theta_{0})\right)}^{2}}=\frac{1}{{\hat{\alpha }}^{H}(\theta_{0}){\hat{R}}^{-1}\hat{\alpha }(\theta_{0})}\\ W^{H}\hat{R}W & =W^{H}(\hat{\sigma^{2}}\hat{\alpha }(\theta_{0}){\hat{\alpha }}^{H}(\theta_{0}))W\\ & =\hat{\sigma^{2}}{\left|W^{H}\hat{\alpha }(\theta_{0})\right|}^{2}=\hat{\sigma^{2}} \end{array}\right.,\\ \hat{\sigma^{2}}=\frac{1}{{\hat{\alpha }}^{H}(\theta_{0}){\hat{R}}^{-1}\hat{\alpha }(\theta_{0})}. \end{array}$$

Furthermore, substituting Equation (8) into Equation (7) yields

(9) $$\left\{\begin{array}{l} \min\alpha^{H}(\theta_{0}){\hat{R}}^{-1}\alpha (\theta_{0}),\\ s.t.{\left(\alpha (\theta_{0})-\hat{\alpha }(\theta_{0})\right)}^{H}C^{-1}(\alpha (\theta_{0})-\hat{\alpha }(\theta_{0}))\leq 1. \end{array}\right.$$

Based on C=εI(ε>0), Equation (9) can be equated by matrix factorization as follows:

(10) $$\left\{\begin{array}{l} \min\alpha^{H}(\theta_{0}){\hat{R}}^{-1}\alpha (\theta_{0}),\\ s.t.{\left\|\alpha (\theta_{0})-\hat{\alpha }(\theta_{0})\right\|}^{2}\leq \varepsilon , \end{array}\right.$$

where ‖⋅‖ denotes the two-parameter operation, and *ε* is the constraint parameter; to avoid ***α***(*θ*_0_) = 0, it should be set as $\varepsilon \leq {\left\|\hat{\alpha }(\theta_{0})\right\|}^{2}$.

Next, assume that the sum of the number of desired signals and the number of interferences is less than the number of array antennas, that is, (*M* + 1) < *N*. Then, a feature decomposition of the covariance matrix of the received snapshots can be obtained by

(11) $$R=\sum_{i=1}^{N}\lambda_{i}u_{i}u_{i}^{H}=U\Lambda U^{H}=U_{S+J}\Lambda_{S+J}{U_{S+J}}^{H}+U_{N}\Lambda_{N}{U_{N}}^{H},$$

where ***U*** is the matrix composed of eigenvectors; ***Λ*** = *diag*{*λ*_1_, *λ*_2_, …, *λ_N_*} is the diagonal matrix composed of the corresponding eigenvalues, and *λ*_1_ ≥ *λ*_2_ ≥ … ≥ *λ_M_*_+1_ ≥ *λ_M_*_+2_ = *λ_M_*_+3_ = … = *λ*_(*N*)_ = *σ_n_*^2^; ***u****_i_* is the eigenvector corresponding to the eigenvalue; ***U****_S+J_* is the signal subspace eigenmatrix; ***Λ****_S+J_* = *diag*{*λ*_1_, *λ*_2_, …, *λ_M_*} is the diagonal matrix consisting of the signal subspace eigenvalues; ***U****_N_* is the noise subspace eigenmatrix; and ***Λ****_N_* = *σ_n_*^2^***I*** is diagonal matrix consisting of the noise subspace eigenvalues.

The right multiplication of ***U****_N_* to (11) yields

(12) $$\begin{array}{cc} RU_{N} & =\left[U_{S+J},U_{N}\right]\Lambda \left[\begin{array}{l} {U^{H}}_{S+J}\\ {U^{H}}_{N} \end{array}\right]U_{N}\\ & =\sigma_{n}^{2}U_{N}. \end{array}$$

Furthermore, multiplying the right side of Equation (2) by ***U****_N_* yields

(13) $$RU_{N}=AR_{S+J}A^{H}U_{N}+\sigma_{n}^{2}U_{N},$$

where ***A*** = {***α***(*θ*_0_), ***α***(*θ*_1_), …, ***α***(*θ_N_*)} is the matrix space of the received signal steering vectors.

According to Equations (12) and (13), it holds that

(14) $$AR_{S+J}A^{H}U_{N}=0,$$

where ***R****_S+J_* is reversible when the signals and interferences are incoherent, so it can be written that

(15) $$A^{H}U_{N}={\left(A^{H}U_{N}\right)}^{H}=0,$$

that is,

(16) $${u_{i}}^{H}\alpha (\theta_{0})=0(i=M+2,\ldots ,N).$$

Furthermore, Equation (16) can be rewritten as follows:

(17) $${\left\|{U_{N}}^{H}\alpha (\theta_{0})\right\|}^{2}=0.$$

Due to the mismatch in the steering vectors, it holds that ${\left\|{U_{N}}^{H}\hat{\alpha }(\theta_{0})\right\|}^{2}\geq 0$, where the equal sign holds if and only if $\hat{\alpha }(\theta_{0})=\alpha (\theta_{0})$. Thus, the steering vector can be corrected based on Equation (10) as follows:

(18) $$\left\{\begin{array}{l} \min\|{{U_{N}}^{H}\alpha (\theta_{0})\|}^{2},\\ s.t.{\left\|\alpha (\theta_{0})-\hat{\alpha }(\theta_{0})\right\|}^{2}\leq \varepsilon . \end{array}\right.$$

The optimal problem defined by Equation (18) can be minimized on the elliptic uncertainty set’s boundary. For computational convenience, in the following, ***α***(*θ*_0_) is abbreviated as ***α***_0_, and $\hat{\alpha }(\theta_{0})$ is abbreviated as ${\hat{\alpha }}_{0}$. The Lagrangian function is defined as follows:

(19) $$f={\left\|U_{N}\alpha_{0}\right\|}^{2}+\lambda ({\left\|\alpha_{0}-{\hat{\alpha }}_{0}\right\|}^{2}-\varepsilon ),$$

where *λ* is a Lagrangian multiplier.

Next, ***α***_0_ can be obtained by taking the partial derivative of Equation (19) as follows:

(20) $$\frac{\partial f}{\partial \alpha_{0}}=2U_{N}{U_{N}}^{H}\alpha_{0}+2\lambda (\alpha_{0}-{\hat{\alpha }}_{0}).$$

Afterward, equating Equation (20) to zero yields

(21) $$\alpha_{0}={\left(\frac{U_{N}{U_{N}}^{H}}{\lambda }+I\right)}^{-1}{\hat{\alpha }}_{0}.$$

Moreover, substituting Equation (21) in the constraints of Equation (18) yields

(22) $${\left\|{\left(\frac{U_{N}{U_{N}}^{H}}{\lambda }+I\right)}^{-1}{\hat{\alpha }}_{0}-{\hat{\alpha }}_{0}\right\|}^{2}=\varepsilon ,$$

where ***I*** can be equated to (***U****_S+J_**U**_S+J_*^H^ + ***U****_N_**U**_N_*^H^), and it can be obtained that

(23) $$\begin{array}{cc} {\left(\frac{U_{N}{U_{N}}^{H}}{\lambda }+I\right)}^{-1} & =\left[U_{N},U_{S+J}\right]\left[\begin{array}{cc} \frac{\lambda }{1+\lambda }I & 0\\ 0 & I \end{array}\right]\left[\begin{array}{c} {U_{N}}^{H}\\ {U_{S+J}}^{H} \end{array}\right]\\ & =\frac{\lambda }{1+\lambda }U_{N}{U_{N}}^{H}+U_{S+J}{U_{S+J}}^{H}. \end{array}$$

Substituting Equation (23) in Equation (22) yields

(24) $${\left\|{\left(\frac{U_{N}{U_{N}}^{H}}{\lambda }+I\right)}^{-1}{\hat{\alpha }}_{0}-{\hat{\alpha }}_{0}\right\|}^{2}={\left\|-\frac{1}{1+\lambda }U_{N}{U_{N}}^{H}{\hat{\alpha }}_{0}\right\|}^{2}.$$

Setting $P=U_{N}{U_{N}}^{H}{\hat{\alpha }}_{0}$ results in $p_{i}=u_{i}{u_{i}}^{H}{\hat{\alpha }}_{0}\left(i=M+2,\ldots ,N\right)$, and Equation (24) can be rewritten as follows:

(25) $$\begin{array}{cc} {\left\|-\frac{1}{1+\lambda }U_{N}{U_{N}}^{H}{\hat{\alpha }}_{0}\right\|}^{2} & ={\left(\frac{1}{1+\lambda }\right)}^{2}\sum_{i=M+2}^{N}{\left|u_{i}{u_{i}}^{H}{\hat{\alpha }}_{0}\right|}^{2}\\ & ={\left(\frac{1}{1+\lambda }\right)}^{2}\sum_{i=M+2}^{N}{\left|p_{i}\right|}^{2}. \end{array}$$

Finally, by substituting Equation (25) into Equation (22), *λ* can be solved as follows:

(26) $$\lambda =\sqrt{\frac{1}{\varepsilon }\sum_{i=M+2}^{N}{\left|p_{i}\right|}^{2}}-1.$$

Next, a value of the constraint parameter *ε* needs to be determined, and combining Equations (21) and (26), an approximate actual steering vector ${\tilde{\alpha }}_{0}$ can be obtained, which is then substituted in Equation (19) to replace ***α***_0_, which can be expressed as follows:

(27) $$\tilde{f}={\left\|U_{N}{\tilde{\alpha }}_{0}\right\|}^{2}+\lambda ({\left\|{\tilde{\alpha }}_{0}-{\hat{\alpha }}_{0}\right\|}^{2}-\varepsilon ).$$

The constraint parameter ε is adjusted so that $\tilde{f}$ gradually approaches the minimum value of *f*_min_; additionally, an optimal constraint parameter *ε*_opt_ corresponding to the assumed desired steering vector ${\hat{\alpha }}_{0}$ is obtained. Substituting *ε*_opt_ into Equation (26) yields

(28) $$\lambda_{\mathrm{opt}}=\sqrt{\frac{1}{\varepsilon_{\mathrm{opt}}}\sum_{i=M+2}^{N}{\left|p_{i}\right|}^{2}}-1.$$

Finally, by substituting Equation (28) into Equation (21), the modified desired steering vector ***α***_0_ can be solved.

### 3.2. Covariance Matrix Reconstruction

Consider the signal with a power *σ*^2^ from an angle *δ* received by an array. Then, the covariance matrix of the received signal can be expressed as follows:

(29) $$R=\sigma^{2}\alpha (\delta )\alpha^{H}(\delta )+\sigma_{n}^{2}I,$$

and its power spectrum is obtained by

(30) $$\begin{array}{cc} P(\delta ) & =\frac{1}{\alpha^{H}(\delta )R^{-1}\alpha (\delta )}=\frac{1}{\alpha^{H}(\delta ){\left(\sigma^{2}\alpha (\delta )\alpha^{H}(\delta )+\sigma_{n}^{2}I\right)}^{-1}\alpha (\delta )}\\ & =\frac{1}{\alpha^{H}(\delta ){\left(\sigma^{2}\alpha (\delta )\alpha^{H}(\delta )\right)}^{-1}\alpha (\delta )}+\frac{1}{\alpha^{H}(\delta ){\left(\sigma_{n}^{2}I\right)}^{-1}\alpha (\delta )}\\ & =\sigma^{2}+\frac{1}{\left(1/\sigma_{n}^{2})(\alpha^{H}(\delta )\alpha (\delta )\right)}=\sigma^{2}+\frac{\sigma_{n}^{2}}{N}, \end{array}$$

where $\frac{\sigma_{n}^{2}}{N}$ denotes the noise power.

In a real-case scenario, the noise power can be estimated by

(31) $${\bar{\sigma }}_{n}^{2}=\frac{1}{K}\sum_{k=1}^{K}\frac{1}{\alpha^{H}(\theta_{k})R^{-1}\alpha (\theta_{k})},$$

where *K* is the number of snapshots, Θ is the angular region other than the desired signals and interferences, *θ* ∈ Θ, and *θ_k_* is the *k*th sampling point in this region.

Then, the actual noise power can be obtained as follows:

(32) $${\hat{\sigma }}_{n}^{2}=N{\bar{\sigma }}_{n}^{2}.$$

To obtain a robust covariance matrix, the noise needs to be removed, and then, the power spectrum is integrated over the angular range Θ*_S_* of the desired signal, which can be expressed as follows:

(33) $$R_{S}=\int_{\Theta_{S}}\left(P\left(\theta \right)-{\bar{\sigma }}_{n}^{2}\right)\alpha \left(\theta \right)\alpha^{H}\left(\theta \right)d\theta .$$

The integral of Equation (33) is positive, so the range of Θ*_S_* is the positive part of $P\left(\theta \right)-{\bar{\sigma }}_{n}^{2}$. Next, substituting Equation (30) into Equation (33) yields

(34) $$R_{S}=\int_{\Theta_{S}}\frac{\alpha (\theta )\alpha^{H}(\theta )}{\alpha^{H}(\theta )R^{-1}\alpha (\theta )}d\theta -\int_{\Theta_{S}}\sigma_{n}^{2}\alpha (\theta )\alpha^{H}(\theta )d\theta .$$

The eigenvalue decomposition of Equation (34) is expressed by

(35) $$R_{S}=\sum_{n=1}^{N}\beta_{n}b_{n}{b_{n}}^{H},$$

where $\beta_{n}$ is the eigenvalue of ***R****_S_*, and ***b****_n_* is the corresponding eigenvector.

Assume a matrix ***B*** = {***b***_1_, ***b***_2_, …, ***b****_N_*}, where ***B***_1_ = {***b***_1_, ***b***_2_, …, ***b****_L_*} is the eigenspace tensored by the first *L* eigenvectors of ***R****_S_*, and ***B***_2_ = {***b****_L_*_+1_, ***b****_L_*_+2_, …, ***b****_N_*} is the eigenspace tensored by the remaining eigenvectors; then, ${\hat{\alpha }}_{0}$ belongs to the subspace ***B***_1_***B***_1_^H^.

Assuming ***Q*** = ***Q***^H^ = ***I*** − ***B***_1_***B***_1_^H^, the desired signal in the received signal can be eliminated using a projection matrix ***Q***^H^ as follows:

(36) $$\begin{array}{cc} X\left(k\right) & =Q^{H}X\left(k\right)\\ & =Q^{H}\left(X_{S}\left(k\right)+X_{J}\left(k\right)+X_{N}\left(k\right)\right)\\ & =Q^{H}X_{J}\left(k\right)+Q^{H}X_{N}\left(k\right). \end{array}$$

Furthermore, substituting Equation (36) into Equation (3) yields

(37) $$\begin{array}{cc} \overset{⏜}{R} & =\frac{1}{K}\sum_{k=1}^{K}Q^{H}X\left(k\right)X^{H}\left(k\right)Q\\ & =Q^{H}\hat{R}Q. \end{array}$$

Additionally, because ***Q***^H^***X***(*k*) = ***Q***^H^***X****_J_*(*k*) + ***Q***^H^***X****_N_*(*k*), Equation (37) can be transformed to

(38) $$\begin{array}{cc} \overset{⏜}{R} & =\frac{1}{K}\sum_{k=1}^{K}Q^{H}X\left(k\right)X^{H}\left(k\right)Q\\ & =\frac{1}{K}\sum_{k=1}^{K}Q^{H}\left[X_{J}\left(k\right)+X_{N}\left(k\right)\right]{\left[X_{J}\left(k\right)+X_{N}\left(k\right)\right]}^{H}Q\\ & =Q^{H}{\hat{R}}_{J}Q+\sigma_{n}^{2}Q^{H}Q. \end{array}$$

After combining Equations (37) and (38), the following expression can be obtained:

(39) $$Q^{H}\hat{R}Q=Q^{H}{\hat{R}}_{J}Q+\sigma_{n}^{2}Q^{H}Q.$$

According to the existing conclusions, the modulus of the steering vectors, which are outside the angular range of the desired signal, does not change with a projection matrix ***Q***^H^ [40]. Following this property, it can be assumed that $Q^{H}{\hat{R}}_{J}Q\approx {\hat{R}}_{J}$, and then, Equation (39) can be transformed to

(40) $${\hat{R}}_{J}\approx Q^{H}\hat{R}Q-\sigma_{n}^{2}Q^{H}Q.$$

However, in practice, a projection matrix ***Q***^H^ cannot completely cancel the desired signal, which results in a biased interference covariance matrix. Therefore, the power spectrum of the received signal is integrated twice to reduce the error. In addition, ***R***^−1^ is replaced by ${\hat{R}}_{J}$, and it is reintegrated over angle Θ*_J_* where the interference is located, which can be expressed by

(41) $$R_{J}=\int_{\Theta_{J}}\frac{\alpha (\theta )\alpha^{H}(\theta )}{\alpha^{H}(\theta ){\hat{R}}_{J}\alpha (\theta )}d\theta .$$

Finally, the corrected interference noise covariance matrix is obtained by

(42) $$R_{J+N}=R_{J}+R_{N}=R_{J}+{\hat{\sigma }}_{n}^{2}I.$$

### 3.3. Proposed Algorithm Workflow

The specific steps of the proposed algorithm are presented in Table 1.

## 4. Simulation Results and Analysis

This section analyzes the performance of adaptive beamforming algorithms through experiments. The conventional Capon algorithm, the IRCB beamforming algorithm [39], and the JMRC algorithm [40] are selected for comparison with the proposed algorithm. The number of elements in the uniform linear array is set to N = 8, with the elements arranged in a uniform linear array with a spacing of half a wavelength. In the experiments, it is assumed that the desired signal, noise, and interference sources are uncorrelated and stationary. There is one desired signal from 0°, and two interference signals from −27° and 13°, respectively. It is assumed that the desired signals have angular mismatches of 5° and 9°, meaning that the directions of the desired signals are estimated to be 5° and 9°, respectively. The simulation parameters, such as SNR and INR, are set as shown in Table 2. All simulation results were obtained through 100 Monte Carlo experiments.

**Figure 1:**
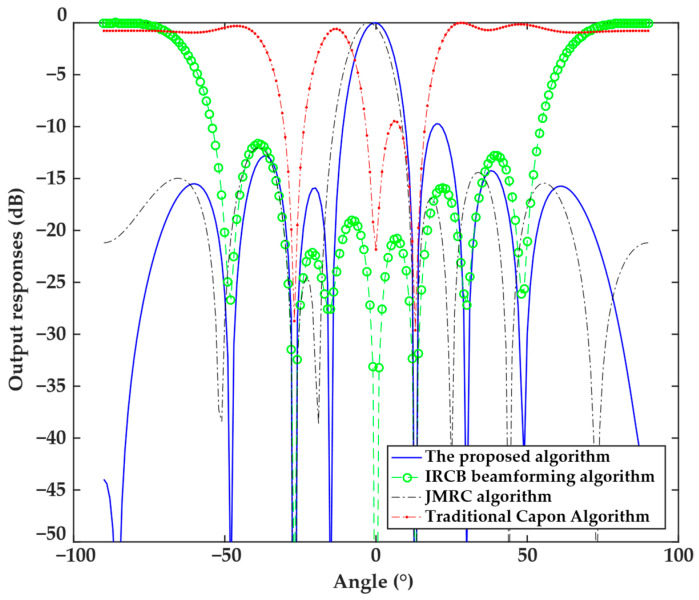
Directional patterns of the four algorithms under the 5° mismatch in the steering vector.

The first experiment analyzed the performance of directional patterns obtained by the proposed algorithms with a 5° mismatch angle of the steering vector. The analysis results are shown in Figure 1, which indicates that due to the steering vector error, the traditional Capon beamforming algorithm and IRCB beamforming algorithm form a null in the angle of the desired signal and thus are not robust, and the traditional Capon beamforming algorithm has an output SINR of −30 dB at the interference angle, with minimal interference attenuation. Hence, both algorithms fail to correct the direction vector of the desired signal, making them susceptible to interference in complex scenarios and lacking robustness. The JMRC algorithm can correct the steering vector error, but the main beam does not strictly align with the desired signal angle, with a deviation of approximately 2.3°, and the directional pattern exhibits noticeable errors. In contrast, the proposed algorithm effectively corrects the mismatch in the steering vector, with the corrected desired signal angle strictly aligned with the desired signal angle. It forms the main beam at the desired signal angle while creating a deep null at the interference angle, with the minimum level of <−50 dB. Therefore, the proposed algorithm not only exhibits robustness against steering vector errors but also adaptively suppresses interference, making its robustness and flexibility superior to other algorithms.

**Figure 2:**
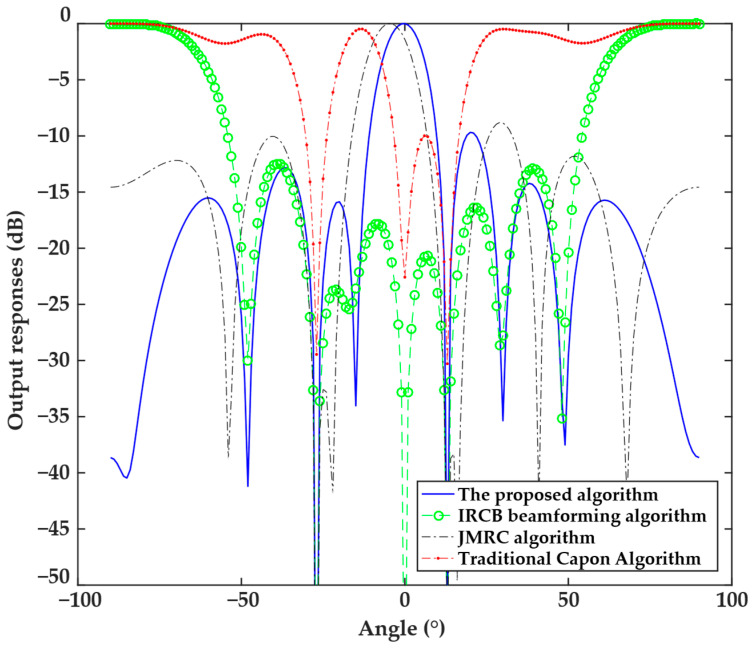
Directional patterns of the four algorithms under the 9° mismatch in the steering vector.

The next experiment analyzed the directional patterns obtained by the four algorithms under the 9° mismatch angle of the steering vector, and the results are illustrated in Figure 2. The results indicate that the traditional Capon beamforming algorithm and IRCB beamforming algorithm form nulls at the angle of the desired signal and are not robust to the steering vector error, making them susceptible to interference in complex scenarios. The directional pattern of the JMRC algorithm shows an obvious shift in the main beam, with a deviation of approximately 6.3°, indicating that the deviation increases as the steering vector error angle increases, making it unable to real-time identify and correct the true desired signal direction. In contrast, the proposed algorithm could effectively correct the mismatch in the steering vector and form the main beam in the direction of the desired signal while generating a depth null in the interference direction. The angle deviation of the desired signal in this experiment was larger than that in the first experiment. Nevertheless, the proposed algorithm could accurately correct the deviation, whereas the performance degradation of the other algorithms was obvious.

**Figure 3:**
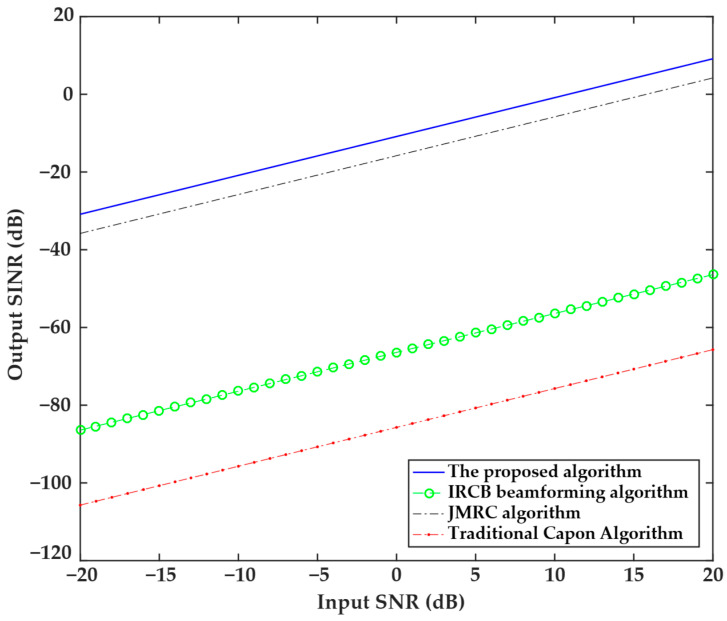
Relationship between the output SINR and input SNR values of the four algorithms.

The third experiment studied the relationship between the output SINR and the input SNR. The experimental setup is as follows: the expected signal direction is fixed at 0°, the number of snapshots is 100, and the input SNR range covers the dynamic range from −20 dB to 20 dB. The results are shown in Figure 3, where it can be seen that all four algorithms showed an increasing trend in the output SINR with the input SNR value. The IRCB beamforming algorithm has an input SNR ranging from −20 dB to 20 dB, with the output SNR slowly increasing from −90 dB to −50 dB. The traditional Capon algorithm has an output SNR that increases linearly with the input SNR, but it has the lowest overall level and the slowest growth rate. Although the JMRC algorithm and the proposed algorithm have similar gain slopes, the proposed algorithm has the largest output SINR value among all algorithms, which is 5 dB greater than the output SINR of the JMRC algorithm. This indicates that the proposed algorithm has strong robustness across a wide SNR range.

**Figure 4:**
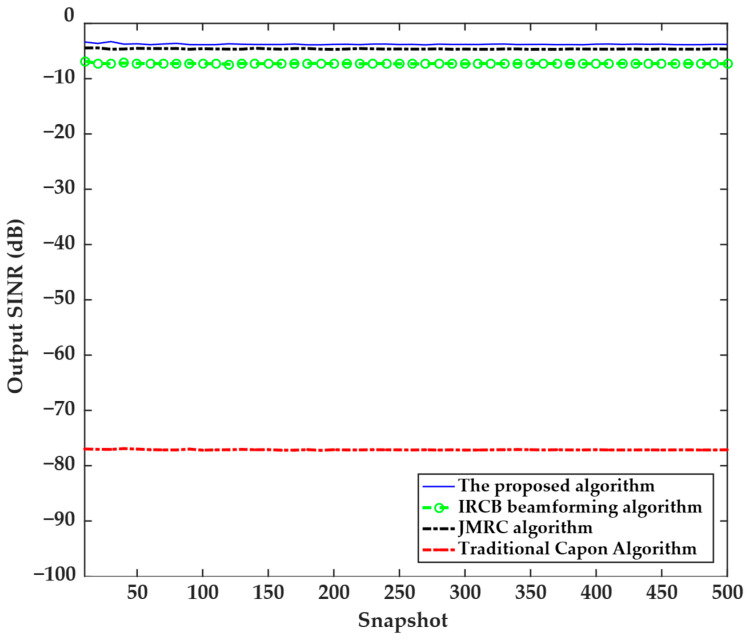
Relationship between the output SINR and the number of snapshots of the four algorithms.

The fourth experiment examined the relationship between the output SINR and the number of snapshots. The experimental parameters are set as follows: the SNR is 10 dB, the interference-to-noise ratio is 20 dB, the interference angle is 70°, and the number of snapshots expanded from 10 to 500. The experimental results are shown in Figure 4, where it can be seen that the four algorithms were not sensitive to the changes in the number of snapshots. The output SINR of the proposed algorithm remains stable within the range of −5 dB to −3 dB, the output SINR of the IRCB beamforming algorithm remains stable within the range of −8 dB to −6 dB, the output SINR of the JMRC algorithm remains stable within the range of −6 dB to −4 dB, and the output SINR of the traditional Capon algorithm remains stable within the range of −78 dB to −76 dB. From this, it can be inferred that the output SINR of the traditional Capon algorithm is significantly lower than that of the other three algorithms, while the output SINR of the proposed algorithm remains the highest among all algorithms. This indicates that the proposed algorithm is more effective and suitable for engineering applications.

**Figure 5:**
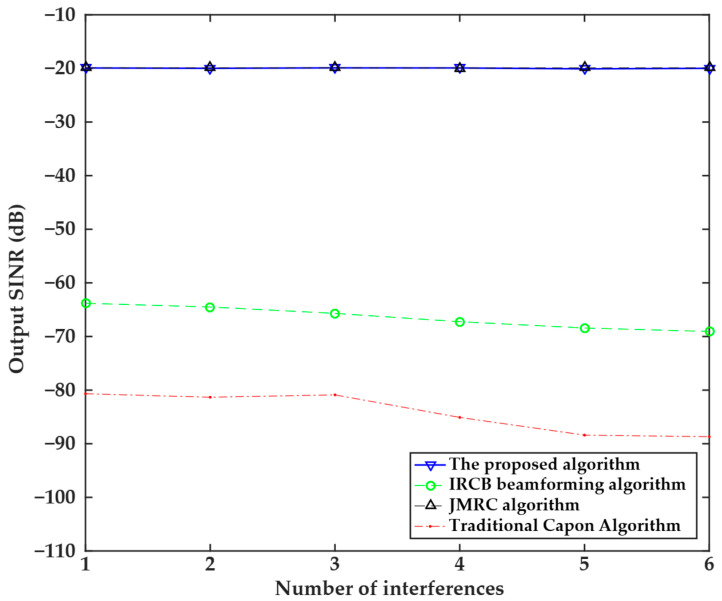
Relationship between the output SINR and the number of interferences of the four algorithms.

The fifth experiment investigated the relationship between the output SINR and the number of interferences, as shown in Figure 5. The experiment used 100 snapshots, and interference angles were set as −30°, −25°, −20°, 20°, 25°, and 30°, respectively. The results indicated that with the increase in the number of interferences, the output SINR values of the four algorithms decreased. The output SINR of the IRCB beamforming algorithm remains stable within the range of −70 dB to −60 dB, but is significantly lower than that of the proposed algorithm due to changes in the number of interferers. The output SINR of the traditional Capon algorithm also decreases slowly with an increase in the number of interferers, dropping from approximately −80 dB to −90 dB, with the lowest output SINR value compared to other algorithms. The proposed algorithm and the JMRC algorithm are insensitive to the number of interference sources. The output SINR of both algorithms remains stable at around −20 dB, with their output SINR values remaining nearly unchanged across all interference source counts. This experiment validates the robust advantages of the proposed algorithm, which maintains a high SINR in multi-interference scenarios, making it the preferred choice for complex interference environments.

**Figure 6:**
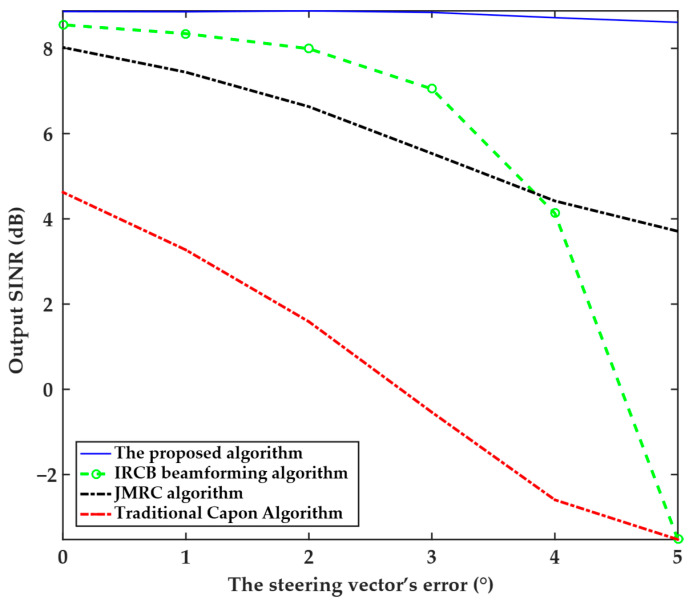
Relationship between the output SINR and the steering vector’s error angle for the four algorithms.

The sixth experiment analyzed the relationship between the output SINR and the steering vector’s mismatch angle, as presented in Figure 6. The experiment is setup with the desired signal incident at 0°, interference from −20°, and the mismatch angle range gradually increasing from 0° to 5°. This set of parameters aims to quantify the impact of array model mismatch on beamforming accuracy and provide data support for error calibration in engineering applications. The experimental results show that the proposed algorithm maintains a stable SINR of approximately 9 dB when the steering vector error ranges from 0° to 5°, with almost no decrease. The IRCB beamforming algorithm maintains an output SINR of 8 dB to 9 dB when the steering vector error is small (0° to 2°); however, when the error exceeds 2°, the output SINR drops sharply (reaching −3 dB at 5°). This indicates that the algorithm has high requirements for steering vector accuracy and necessitates precise estimation of the target direction; otherwise, its mismatch tolerance capability drops sharply. The output SINR of the JMRC algorithm decreases slowly and linearly with steering vector error, from 8 dB at 0° to 4 dB at 5°. Although performance decreases with error, the slow linear decrease indicates that the JMRC algorithm has some robustness to steering vector mismatch. However, the overall output SINR level is lower than that of the proposed algorithm, and its mismatch tolerance is weaker than that of the proposed algorithm. The output SINR of the traditional Capon algorithm decreases rapidly and linearly with steering vector error, from 5 dB at 0° to −3 dB at 5°, indicating that it is most sensitive to steering vector error. The proposed algorithm maintains an extremely high and stable output SINR as the mismatch angle increases, indicating its excellent performance in steering vector mismatch correction and strong robustness. Other algorithms are limited by steering vector errors and exhibit weaker robustness.

According to the analysis results of the above-presented experiments, in the case of a steering vector mismatch, the proposed algorithm could correct the steering vector of the desired signal and ensure the main lobe of the directional pattern was aligned with the desired signal direction. Moreover, the proposed algorithm could adaptively form a null in the interference angle and effectively suppress the interference, improving the output SINR. Consequently, the performance of the proposed algorithm was better than that of the other algorithms. The performance comparison of steering vector correction and output SINR among the proposed algorithm, IRCB beamforming algorithm, JMRC algorithm, and traditional Capon algorithm is given in Table 3.

Finally, we compared the computational complexity of algorithms. The traditional Capon algorithm complexity is *O*(*N*^3^) (*N* is the number of array antennas), which involves only matrix inverse and multiplication operations. The proposed algorithm includes the following steps: eigenvalue decomposition, projection matrix construction, power spectrum integration, and quadratic integration correction. The overall complexity is *O*(max(*LN*^3^, *KN*^2^)). *L* represents the number of angle sampling points, and the quadratic integration step introduces O(*LN*^3^) complexity; *K* represents the number of snapshots. Although the theoretical complexity of the proposed algorithm is higher than that of the traditional Capon algorithm, its performance advantages in terms of mismatch resistance, interference resistance, and low SNR scenarios have significant engineering value.

## 5. Conclusions

In this study, the received signal covariance matrix was decomposed into signal and noise spaces using the conventional Capon beamforming algorithm. In addition, based on the orthogonality between the desired signal steering vector and the noise space, the desired signal in the received signal was removed. Furthermore, the constraint parameter was adjusted dynamically, and the estimated desired signal steering vector was corrected. Combined with the covariance reconstruction, the interference covariance was obtained by reintegrating the angle where the interference signal was located, and the optimal filtering weight vector was obtained. The performance of the proposed algorithm is validated through simulation experiments and compared with three existing algorithms. The simulation results show that the proposed algorithm exhibits negligible performance degradation within a mismatch angle of ±9°, outperforming the three existing algorithms. Furthermore, the proposed algorithm achieves the highest output SINR under various conditions, including adaptive input SNR (−20 dB to 20 dB), wide snapshot requirements (10–500), and dense scenarios with multiple interferers (1–6). When the input SNR is the same, the output SINR of proposed algorithm is 5 dB higher than that of the JMRC algorithm. When the number of snapshots is the same, the output SINR of proposed algorithm is on average 2 dB higher than that of the JMRC algorithm. As the number of interferences increases gradually, the output SINR of the proposed algorithm remains consistently high. Comprehensive validation shows that the proposed algorithm demonstrates strong robustness in four key areas: beamforming mismatch correction, wide dynamic range adaptability, low snapshots stability, and multi-interference suppression. Under identical conditions, it achieves a higher output SINR than the other three algorithms, making it the optimal anti-interference solution in complex electromagnetic environments. However, the computational complexity of the proposed algorithm is theoretically higher than that of the traditional Capon algorithm. Although the complexity is increased, its performance advantages in terms of mismatch resistance, anti-jamming capability, and low SNR scenarios possess significant engineering application value, which can compensate for the increase in complexity in specific scenarios. Meanwhile, although this study validated the algorithm’s superiority in complex electromagnetic environments via high-fidelity simulation, empirical verification on an SDR platform remains the top priority for future work.

## Figures and Tables

**Table 1:** Steps in the robust capon beam formation algorithm.

Algorithm	Description
Input	Receive signal: ***X***(*t*); Initialization constraint parameter: *ε*.
Step 1	Find the covariance matrix *R* of the received signal *X*(*t*), then perform an eigenvalue decomposition to obtain its noise subspace matrix ***U****_N_*: $R=E[X^{H}(t)X(t)]=U_{S+J}\Lambda_{S+J}{U_{S+J}}^{H}+U_{N}\Lambda_{N}{U_{N}}^{H};$
Step 2	The steering vector *α*_0_ and the Lagrange multiplier *λ* are derived as follows: $\alpha_{0}={\left(\frac{U_{N}{U_{N}}^{H}}{\lambda }+I\right)}^{-1}{\hat{\alpha }}_{0}\;\mathrm{and}\;\lambda =\sqrt{\frac{1}{\varepsilon }\sum_{i=M+2}^{N}{\left|p_{i}\right|}^{2}}-1;$
Step 3	Substituting this *α*_0_ and *λ* into Equation (19) yields the Lagrangian function: $\tilde{f}={\left\|U_{N}{\tilde{\alpha }}_{0}\right\|}^{2}+\lambda ({\left\|{\tilde{\alpha }}_{0}-{\hat{\alpha }}_{0}\right\|}^{2}-\varepsilon );$
Step 4	By adjusting the constraint parameter *ε*, the value of $\tilde{f}$ in Equation (27) is gradually made to approach its minimum value *f*_min_. In this way, the optimal constraint parameter *ε*_opt_ corresponding to *f*_min_ is obtained;
Step 5	After substituting *ε*_opt_ into Equation (26) to obtain $\lambda_{\mathrm{opt}}=\sqrt{\frac{1}{\varepsilon_{\mathrm{opt}}}\sum_{i=M+2}^{N}{\left|p_{i}\right|}^{2}}-1$. substituting λ_opt_ into Equation (21) yields the modified desired orientation vector $\alpha_{0}={\left(\frac{U_{N}{U_{N}}^{H}}{\lambda_{\mathrm{opt}}}+I\right)}^{-1}{\hat{\alpha }}_{0};$
Step 6	Integration of the power spectrum over the desired signal angle range Θ*_S_* removes the effect of noise (Equation (34)): $R_{S}=\int_{\Theta_{S}}\frac{\alpha (\theta )\alpha^{H}(\theta )}{\alpha^{H}(\theta )R^{-1}\alpha (\theta )}d\theta -\int_{\Theta_{S}}\sigma_{n}^{2}\alpha (\theta )\alpha^{H}(\theta )d\theta ,$ and eigenvalue decomposition of the integration result (Equation (35)): $R_{S}=\sum_{n=1}^{N}\beta_{n}b_{n}{b_{n}}^{H};$
Step 7	Construct the projection matrix ***Q*** based on $\overset{⏜}{R}=Q^{H}{\hat{R}}_{J}Q+\sigma_{n}^{2}Q^{H}Q$. Combine Equations (39) and (40) to derive the interference covariance matrix ${\hat{R}}_{J}\approx Q^{H}\hat{R}Q-\sigma_{n}^{2}Q^{H}Q;$
Step 8	Perform a double integral on the angular region Θ*_J_* of the interference signal, replace the inverse matrix with the integration result: $R_{J}=\int_{\Theta_{J}}\frac{\alpha (\theta )\alpha^{H}(\theta )}{\alpha^{H}(\theta ){\hat{R}}_{J}\alpha (\theta )}d\theta ,$ and correct the interference noise covariance matrix ***R****_J+N_* (Equations (41) and (42)): $R_{J+N}=R_{J}+R_{N}=R_{J}+{\hat{\sigma }}_{n}^{2}I;$
Step 9	Substitute the corrected desired signal steering vector *α*_0_ and the corrected interference-plus-noise covariance matrix ***R****_J+N_* into equation to obtain the optimal weight vector $W_{\mathrm{opt}}=\frac{R_{J+N}^{-1}\alpha_{0}}{\alpha_{0}^{H}R_{J+N}^{-1}\alpha_{0}}.$
Output	Optimal weight vector: ***W***_opt_.

**Table 2:** Simulation parameters of the test system and signal model.

Parameter	Value
Number of array elements	8
Formation type	Uniform array
Desired signal direction	0°
Mismatch angles	5°, 9°
Angles of interference	−27°, 13°
Number of snapshots	512
SNR	20 dB
INR	20 dB

**Table 3:** Performance comparison.

	The Proposed Algorithm	IRCB Beamforming Algorithm [39]	JMRC Algorithm [40]	Traditional Capon Algorithm
Steering vector mismatch robustness	Main lobe strictly aligned (0° deviation)	Form a null in the angle of the desired signal	Main lobe deviation of 2.3°	Form a null in the angle of the desired signal
Wide SNR adaptive	The output SINR increases with input SNR, and SINR ≥ −30 dB	The output SINR increases with input SNR, and SINR ≥ −90 dB	The output SINR increases with input SNR, and SINR ≥ −35 dB	The output SINR increases with input SNR, and SINR ≥ −108 dB
Snapshot number robustness	The output SINR remains constant as −4 dB for different snapshot counts.	The output SINR remains constant as −7 dB for different snapshot counts.	The output SINR remains constant as −5 dB for different snapshot counts.	The output SINR remains constant as −77 dB for different snapshot counts.
Output SINR with multiple interference	≈−20 dB	−70 dB to −60 dB	≈−20 dB	−90 dB to −80 dB
Output SINR with mismatch angle increasing	≈9 dB	−3.5 dB to 8.5 dB	3.8 dB to 8 dB	−3.5 dB to 4.5 dB

## Data Availability

Data is contained within the article.
